# Recombinant Live-Attenuated *Salmonella* Vaccine for Veterinary Use

**DOI:** 10.3390/vaccines12121319

**Published:** 2024-11-26

**Authors:** Domitila Brzoskowski Chagas, Francisco Denis Souza Santos, Natasha Rodrigues de Oliveira, Thaís Larré Oliveira Bohn, Odir Antônio Dellagostin

**Affiliations:** 1 Núcleo de Biotecnologia, Centro de Desenvolvimento Tecnológico, Universidade Federal de Pelotas, Pelotas 96010-610, Rio Grande do Sul, Brazilthais.larre@ufpel.edu.br (T.L.O.B.); 2 Faculdade de Medicina, Universidade Federal do Rio Grande, Rio Grande 96200-400, Rio Grande do Sul, Brazil

**Keywords:** vaccine development, *Salmonella*, attenuation, RASV, animal

## Abstract

Vaccination is essential for maintaining animal health, with priority placed on safety and cost effectiveness in veterinary use. The development of recombinant live-attenuated *Salmonella* vaccines (RASVs) has enabled the construction of balanced lethal systems, ensuring the stability of plasmid vectors encoding protective antigens post-immunization. These vaccines are particularly suitable for production animals, providing long-term immunity against a range of bacterial, viral, and parasitic pathogens. This review summarizes the progress made in this field, with a focus on clinical trials demonstrating the efficacy and commercial potential of RASVs in veterinary medicine.

## 1. Introduction

The growing human population has led to an increase in the demand for foods of animal origin, resulting in consequent changes in agricultural production [1]. Animals are often produced in confinement, which makes them vulnerable to the spread of various diseases [2]. Many infectious microorganisms can be zoonotic and are transmitted from animals—whether they are food animals, companion animals, or wildlife—to humans [3,4]. Furthermore, these diseases affect the well-being and productivity of animals, causing economic losses and impacting the food supply [5].

There are solutions that can help control diseases in production animal farms, including effective sanitation in facilities; the quarantine or slaughter of contaminated animals; the administration of treatment with antibiotics, antivirals, and antiparasitics; and vaccination [2]. However, there are growing concerns related to antibiotic resistance associated with their extensive use [6,7]. This is coupled with the high cost of developing new, effective, non-toxic drugs to treat bacterial, viral, and parasitic infections [8]. These challenges have become significant obstacles for the treating of infectious diseases.

Therefore, vaccination has emerged as an alternative that can reduce the reliance on antibiotics for treating infections [1]. While much of the current research has focused on recombinant subunit vaccines, the cost of veterinary vaccines is primarily due to the purification process required for these proteins, which makes them more expensive compared to traditional vaccines [9]. Despite their lower production costs, bacterins have been associated with variable efficacy and potential side effects, as observed in leptospirosis [10]. While each vaccination strategy has its own set of advantages and disadvantages, veterinary vaccines must prioritize safety, cost effectiveness, and the ability to provide long-lasting protective immunity against pathogenic microorganisms [8].

Among the various vaccine formulations using infectious bacterial agents as carriers of immunoprotective antigens, *Salmonella* stands out. This enteropathogenic bacterium infects both humans and animals, causing a variety of illnesses from gastroenteritis to systemic typhoid fever [11,12]. The prevalence of *Salmonella* varies geographically, with *Salmonella enterica* serovar Typhimurium (*S.* Typhimurium) being one of the most commonly isolated serovars from animals and humans worldwide. Some serovars exhibit host specificity, such as *Salmonella enterica* serovar Choleraesuis (*S.* Cholerasuis) in pigs, *Salmonella enterica* serovar Abortusovis (*S.* Abortusovis) in sheep, and *Salmonella enterica* serovar Dublin (*S.* Dublin) in cattle [13,14,15].

Despite its potential pathogenicity, *Salmonella* is easily managed and genetically manipulated, making it an ideal candidate for antigen delivery systems for several reasons [16]. The inactivation of metabolic genes in live vaccines enables the expression of essential antigens and virulence factors while constraining their ability to proliferate. Consequently, vaccine strains engineered and deleted via techniques such as site-directed mutagenesis demonstrate complete biological containment [17,18].

These attenuated strains replicate the natural infection process, possessing the ability to invade and replicate within mucosa-associated lymphatic tissues (MALT) and gut-associated lymphatic tissues (GALT), such as Peyer’s patches, before spreading systemically via mesenteric lymph nodes [18,19]. This characteristic dissemination pattern enables *Salmonella* to evoke robust protective immunity, particularly when administered orally, which stimulates both mucosal and systemic immune responses [20,21]. Additionally, several studies have shown that administering live-attenuated *Salmonella* vaccines via intramuscular or subcutaneous routes in animals (such as mice, dogs, and goats) effectively stimulates an immune response [22,23,24].

The adoption of rationally live-attenuated *Salmonella* vaccines is already underway in farm animal vaccination programs, which aim to control infections and prevent disease spread [25,26]. Additionally, numerous studies have explored vaccine constructs based on live-attenuated *Salmonella*, primarily carrying antigens against a variety of other organisms, including bacteria, viruses, and parasites [27,28,29,30,31]. These vaccines are preferably administered orally and nasally because of their ease of delivery, allowing for needle-free administration in a straightforward and painless manner. They can be administered via spray or mixed into water, making them more suitable for widespread use in commercial animals such as poultry, swine, and fish [31,32]. However, oral administration in ruminants still poses challenges due to the process of rumination and regurgitation of food, necessitating techniques like microparticle encapsulation to enhance efficacy [33].

**Figure 1:**
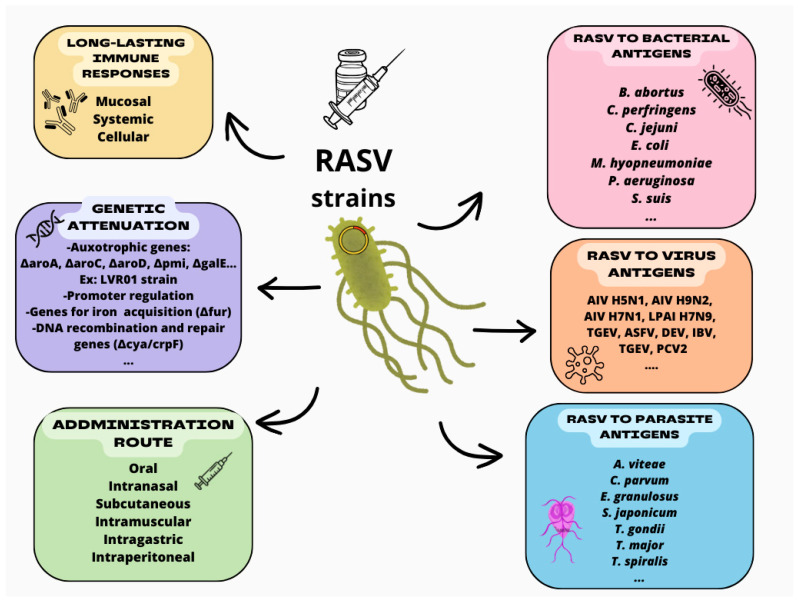
Recombinant live-attenuated *Salmonella* vaccines (RASV): main characteristics and applications as veterinary vaccines for bacterial, viral, and parasitic antigens. AIV, avian influenza viruses; LPAI, low pathogenic avian influenza; TGEV, transmissible gastroenteritis virus; ASFV, African swine fever virus; DEV, duck enteritis virus; IBV, infectious bronchitis virus; PCV2, porcine circovirus type 2.

The significance of using live-attenuated *Salmonella* vaccines in veterinary practices is reinforced by their storage convenience [16]. They can be easily freeze-dried and maintained at room temperature, offering a notable advantage for their implementation in livestock, especially in regions lacking vaccine refrigeration facilities [33,34]. Emphasizing these diverse benefits, this review examines compelling findings from past and ongoing studies that explore the progress of recombinant live-attenuated *Salmonella* vaccines (RASVs) as a versatile delivery vector (Figure 1) for the prevention of various infectious diseases.

## 2. Commercial Veterinary Vaccines Against *Salmonella* in Farm Animals

The use of vaccines to control infections caused by *Salmonella* spp. has been widely adopted in various countries [35]. However, the use of commercial bacterins poses a risk of heightened vaccine reactions at the injection site, typically following intramuscular administration, because of the presence of toxic components in bacterial cells, particularly lipopolysaccharides (LPS) and oil emulsion adjuvants [36]. Additionally, many killed whole-cell vaccines offer limited cross-protection against other antigenically related serotypes [11,14].

Live-attenuated *Salmonella* vaccines, which are designed to decrease disease prevalence and confer protection against various pathogen strains, are becoming increasingly commercially available [37,38]. These vaccines are being licensed for use in production animals across several countries [39]. Among the live-attenuated vaccines against *Salmonella* available in Europe and Australia for chicken producers are those targeting *S*. Typhimurium, *S.* Enteritidis, and *S.* Gallinarum. In a field study, Lyimu et al. [25] evaluated the effect of three commercial live-attenuated vaccine strains on cecal immune genes and compared with cytokine expression. The vaccine induced more anti-inflammatory cecal environment and Th1 responses, crucial to limiting *Salmonella* contamination in chickens. Furthermore, they reported an increase in serum IgG in the vaccine group that received the commercial vaccine against *S.* Typhimurium when compared to the control. However, they also reported that the live vaccine can modify the shape of different microbiota profiles.

Another example of commercially available vaccines for veterinary use is the live-attenuated vaccine against *S.* Typhimurium in pigs [26]. The *Salmonella* vaccine has already been evaluated in sows and piglets weaned from four, three, or twenty-four days to six or seven weeks of age [40,41] and in sows and gilts [42]. Recently, in a study using different swine production cycles, the use of the vaccine in sows, piglets, and fattening pigs resulted in control over *S.* Typhimurium infections and decreased prevalence of positive lymph nodes at slaughter [26].

## 3. Role of Attenuation in the Recombinant *Salmonella* Vaccine

*Salmonella* has been used as a vector for heterologous antigens, mainly because of its ability to elicit long-lasting mucosal, systemic, and cellular immune responses [17]. It can be administered via various routes, including oral, nasal, subcutaneous, and intramuscular administration [20]. However, for the use of live recombinant *Salmonella*, the attenuation of virulence factors is mandatory in order to prevent unwanted side effects such as fever and diarrhea [43].

Several approaches have been studied for the development of live-attenuated recombinant *Salmonella* vaccines, including different mutations that guarantee the attenuation of these strains through regulated delayed attenuation, delayed antigen synthesis, and/or delayed lysis [8,17]. These strategies have enabled the construction of a balanced lethal system and the stability of plasmid vectors encoding protective antigens in vivo after immunization [17,44].

Regulation allows these vectors to present characteristics similar to those of the wild type, enabling their survival and transit through the gastrointestinal tract and the execution of the initial stages of infection before exhibiting attenuation [45]. These studies have led to advances and discoveries in biological containment and antigen delivery systems using *Salmonella* [20]. The deletion of Δpmi or ΔgalE genes makes the strains dependent on exogenous mannose and galactose, respectively [46]. These genes encode surface antigens, such as O antigen side chains, and cause phenotypic changes in lipopolysaccharides, which are crucial factors for host colonization [18]. Furthermore, it is possible to utilize the deletion or mutation of genes necessary for the biosynthesis of metabolically essential elements, such as aromatic amino acids and vitamins, including ΔaroA, ΔaroC, and ΔaroD deletions [47]. An example of this type of attenuation that has been used in a study is the *S.* Typhimurium LVR01 strain, which was constructed by introducing a null deletion in the ΔaroC gene of the canine parental isolate of *S.* Typhimurium, P228067 [48].

Likewise, it is possible to regulate expression at or through the promoters of the chromosomal repressor gene *lacI* in regulatory pathways with pleiotropic effects (*cya*, *crp*, *phoP*). This can include using activating or repressor protein binding sequences for genes of iron acquisition (Δ*fur*), encoding the regulatory system of virulence components (Δ*phoP* and Δ*phoQ*), or including mutations in DNA recombination and repair genes (Δ*cya*/*crpF*) or in the *cAMP* receptor protein (Δ*crp*), which is regulated via an *araC* P BAD cassette [47]. Thus, the expression of these genes is regulated by arabinose or mannose supplementation, occurring only during in vitro growth [17]. Following the colonization of lymphoid tissues, synthesis of the associated proteins ceases due to the absence of mannose or arabinose in vivo [28]. Consequently, attenuation develops progressively in vivo, inhibiting the onset of disease symptoms and inducing the desired antigen-specific immune response.

Moreover, regulated delayed lysis in vivo prevents RASV persistence, aiding biocontainment by ensuring the death of the bacteria following the colonization of immune tissues [17,47]. The system regulates the expression of enzymes required for synthesizing two key components of the cell wall’s peptidoglycan layer: diaminopimelic acid (DAP) and muramic acid. DAP synthesis is controlled by aspartate semialdehyde dehydrogenase (encoded by *asd*), while UDP-N-acetylglucosamine enolpyruvyl transferase (encoded by *murA*) regulates muramic acid production [44]. The expression of these enzymes is engineered to be regulated by exogenous arabinose; in its absence, cell lysis occurs due to an inability to synthesize the cell wall [49].

Another crucial aspect of a vaccine vector containing heterologous antigens is the stability of the plasmid [8]. Regulation of the levels and location of expression of these antigens can have a significant impact on the immunogenicity of the vaccine, potentially reducing colonization capacity and, consequently, immunological efficacy [18]. To address the problem of instability in the chromosomal integration of foreign genes, systems have been developed that include the integration of the foreign gene into the bacterial chromosome, the optimization of heterologous antigen codons [50], the use of inducible promoters in vivo [49], and those by which DNA encodes the foreign gene in a suicide vector [44].

Thus, vaccine strains generally incorporate more than one mutation or deletion in genetic constructs, making it possible to eliminate essential genes involved in virulence regulatory systems [45]. This ensures attenuation, prevents virulence reversal, and eliminates potential side effects [51]. Furthermore, these deletions increase the colonization capacity, survival in the mucosal environment, and immunogenicity of RASV vaccine constructs [20,47].

## 4. Molecular Mechanisms of Immune Stimulation by Recombinant *Salmonella*

Live-attenuated *Salmonella* strains used as vaccine delivery vehicles for heterologous antigens should effectively cross the epithelial barrier, reach the underlying antigen-presenting cells in the MALT, and trigger a strong immune response [51]. These mutants establish a limited infection in the host, and during this harmless infection, they deliver a variety of in vivo synthesized antigens directly to B and T lymphocytes in the GALT [52,53].

Typically, the antigens presented by RASV colonize internal effector lymphoid tissues without compromising protective functions or integrity. The interaction between *Salmonella* and its host is initiated by various virulence factors, including type III secretion systems associated with *Salmonella* pathogenicity islands 1 and 2 (SPI-1 and SPI-2) [8,52,54]. The SPI-1-encoded system enables *Salmonella* to invade various lymphoid tissues associated with the intestinal, nasopharyngeal, and bronchial mucosa. Bacterial internalization triggers alterations in host cell signaling pathways, impacting essential cellular processes such as membrane trafficking, cell division, antigen presentation, and cytokine production [51].

*Salmonella*, having adapted to the mucosal surface environment, begins the infection process, remaining in membrane-bound vacuoles, and once it reaches the mesenteric lymph nodes in antigen-presenting cells, they produce recombinant proteins [51]. Antigen delivery results in a generalized immune response that targets intestinal sensory cells, known as Peyer’s patch M cells [44]. These cells play a key role in stimulating mucosal immune responses [55]. Furthermore, *Salmonella* spp. can be taken up by phagocytic cells and cross the reticulo-endothelial system, thereby stimulating systemic immune responses [56]. *Salmonella* efficiently targets MALT and induces local and systemic immunity [12,57]. Dendritic cells, neutrophils, and macrophages are activated in response to antigens being present in the MALT, having recognized pathogen-associated molecular patterns (PAMPs) and endogenous danger-associated molecular patterns (DAMPs), such as T3SS-1, fimbriae, and other bacterial surface adhesins [56].

Protein antigens are processed and presented through the major histocompatibility complex (MHC), stimulating T cell responses [55]. The signaling and activation of phagocytic cells initiate a critical immune response that establishes links between the innate and adaptive immune systems [58]. *Salmonella* has a preference for residing within macrophages, where the activation of these cells by interferon gamma (IFN-γ) produced by Th1 cells significantly contributes to bacterial elimination [54]. IFN-γ, commonly referred to as macrophage activating factor (MAF), affects the duration of macrophage activation and is crucial during infection. The secretion of IFN-γ is dependent on IL-18, also known as the IFN-γ-inducing factor, and is vital for establishing early host resistance to *Salmonella* infection [59]. During primary and secondary infection, *Salmonella* is dependent on IL-12, IFN-γ, and tumor necrosis factor α (TNF-α).

Classical activation by bacterial LPS or IFN-γ modifies the cells’ secretory profile by promoting the production of organic nitrate compounds, including nitric oxide (NO) [56]. Mucosal DC responses to inflammatory stimuli may enhance their capacity to preselect antigens expressed by recombinant *Salmonella* for targeting specific T and B cells [60]. Alternative activation by IL-4, IL-10, or IL-13 promotes the production of polyamines and proline, stimulating cell proliferation. The presence of *Salmonella* in these cells triggers cytokine secretion, leading to an inflammatory response or programmed cell death via apoptosis [56].

The RASV consistently produces recombinant protein for an appropriate duration under SPI-2-regulated conditions, after which it is translocated into the cytosol via the SPI-2 T3SS [61]. Secreted peptides are processed and presented to major histocompatibility complex (MHC) class I and II molecules to stimulate T cell responses [56]. Immunization with attenuated vaccines is a safe and effective method to induce the production of both serum and mucosal antibodies against the *Salmonella* carrier, as well as foreign antigens [12,62].

## 5. *Salmonella* as a Vaccine Vector Against Different Pathogens

Attenuated vaccines are produced using live, whole bacterial cells or viral particles that have been subjected to in vitro passages, chemical treatments, or genetic manipulation to obtain strains lacking virulence in the host. Despite the loss of virulence, these attenuated strains retain their ability to provoke a strong immune response, closely mimicking natural infection [63]. Although several conventional attenuated vaccines are available for veterinary use, concerns about the risk of residual virulence, particularly in immunocompromised hosts, and higher sensitivity to cold chain disruptions have driven the search for safer options [9,63].

Studies in mice vaccinated with a live attenuated *Salmonella* delivering *E. coli* antigens [64,65] to control post-weaning diarrhea in swine indicated that vaccine constructs were not detected in the feces of immunized animals for up to three weeks post-oral inoculation, suggesting minimal or no environmental excretion. Another study using attenuated *S. Typhimurium* carrying a DNA vaccine against *Streptococcus agalactiae* in fish showed that the bacteria were eliminated from the tissues four weeks post-immunization [32]. Conversely, Jiang et al. [66] observed that oral vaccination with strains χ9241-tHP and χ9352-tHP resulted in high (10^4^ to 10^7^ CFU/g) and moderate (10^2^ to 10^4^ CFU/g) levels of recombinant *Salmonella* shedding in fecal samples from chickens at 14 and 11 days of age, respectively. Similarly, rectal swabs from pigs orally vaccinated with C500 variants of *S. Choleraesuis* showed variable levels of fecal shedding [67]. These results highlight the need to test different vectors to achieve an optimal balance among immunogenicity, stability, and biocontainment.

The effective immune response and protection induced by live-attenuated recombinant *Salmonella* has been described in several studies using parasitic, bacterial, and viral antigens. The reviewed articles are compiled and presented in a detailed list including the heterologous bacterial, parasitic and viral antigens, respectively expressed in different strains of *Salmonella*, as well as the model of attenuation used, route and dose administered, immune response induced, and animal model used. This is a summary of information published to date regarding the use of recombinant attenuated *Salmonella* vaccine (RASV) as a vaccine vector. The potential of RASV is evident, and it requires further exploration.

### 5.1. RASV Administration Routes

In our review, we found that mucosal administration—primarily via the oral route—is more frequently explored for RASV than parenteral routes, such as intramuscular, subcutaneous, or intraperitoneal injection. A limited number of studies have used intranasal [31,68,69,70,71] and intragastric routes [32,72,73,74]. The choice of administration route can impact antigen immunogenicity, influencing immune cell priming (especially antigen-presenting cells) and promoting both local and systemic immunity [75]. Mucosal routes may be more effective, as most infectious agents enter through mucosal surfaces. Vaccination through mucosal routes can stimulate protective responses at these surfaces and systemically [76]. Conversely, subcutaneous and intramuscular vaccines form antigen depots at the injection site, attracting APCs to migrate to nearby lymph nodes, where they activate the immune response [76].

Several studies have evaluated RASV administration routes in animal models. Lalsiamthara et al. [77] examined the efficacy of an RASV containing four *Brucella* antigens administered via parenteral routes (intraperitoneal, intramuscular, and subcutaneous) in mice. The parenteral administration showed higher protection rates against *B. abortus* compared to oral administration. Another study compared intraperitoneal and oral administration of an *S.* Typhimurium vaccine expressing *B. abortus* antigens in mice. The intraperitoneal route induced stronger humoral (IgG levels) and cellular (TNF-α and IFN-γ) responses, with greater protection against *B. abortus* infection post-challenge [29].

In contrast, Cong et al. [73] demonstrated the potential of mucosal routes for an RASV expressing *T. gondii* antigens. The vaccine was administered to mice orally, nasally, or intramuscularly. Mice vaccinated via mucosal routes (oral and nasal) showed higher IgG and IgA levels than those vaccinated intramuscularly. Intranasal vaccination stimulated greater CD4^+^ and CD8^+^ T-cell activation, with cytokine levels (IFN-γ and IL-2) also being higher in orally and nasally vaccinated mice. Survival rates post-challenge were 60% for oral, 40% for intranasal, and 20% for intramuscular routes.

Another study by Hyoung et al. [70] evaluated an attenuated *S.* Typhimurium mutant expressing hemagglutinin from avian influenza viruses (H7N3, H7N7, and H7N9) in chickens via intramuscular, nasal, or oral routes. The RASV induced strong humoral (neutralizing antibodies) and cellular (IFN-γ, IL-17, and IL-10) responses across all routes, with immunization conferring significant protection against a subsequent challenge with the H7N1 virus. These findings suggest that RASVs can effectively elicit immune responses across multiple administration routes, providing flexibility in tailoring vaccine strategies based on the pathogen and target population.

### 5.2. Recombinant Salmonella Expressing Bacterial Antigens

Several studies were reviewed and are summarized in Table 1, which presents the different *Salmonella* strains, routes, doses, animal models, and the types of immune response stimulated. In one study [55], an RASV containing chromosomal fusion genes that encode the secretion signal for the SPI-2 effector protein, SspH2, and the pathogenic outer membrane lipoprotein from *Leptospira*, LipL32, was administered orally to rats. The animals received a dose of 1 × 10^7^ colony forming units (CFU) per mouse of different strains of *S*. Typhimurium (ST) or saline (PBS) alone on days 0, 14, and 28. After vaccination, the group that received the RASV exhibited significantly elevated titers of total immunoglobulin G (IgG) and immunoglobulin A (IgA) specific to the rLipL32 protein, with detectable levels persisting up to 77 days post-vaccination. Notably, following the third immunization on day 28, the anti-LipL32 antibody titers in mice immunized with the RASV were significantly higher than those in mice that received only PBS (*p* < 0.05). Additionally, to assess the cellular immune response, there was a significant increase in the production of the LipL32-specific cytokines IFN-γ and IL-4 in splenocytes from mice vaccinated with the RASV compared to the control group treated with PBS (*p* < 0.05) [55].

Another RASV using *Salmonella* Typhimurium that expresses the R2 antigen of NrdF from *Mycoplasma hyopneumoniae* was used to orally inoculate mice at a dose of 10^9^ CFU, followed by two more boosters at the same dose. In this study, a mucosal IgA-type immune response was elicited in lung washings, but a significant level of NrdF-specific serum IgG was not detected [78]. Interestingly, Chen et al. [80] demonstrated that a DNA vaccine with a eukaryotic expression plasmid, encoding the *M. hyopneumoniae* NrdF antigen through an *S.* Typhimurium live-attenuated *aroA*, when administered orally to mice at a dose of 2 × 10^8^ CFU followed by a booster dose of 3 × 10^8^ CFU, induced significant NrdF-specific IFN-γ production. However, mice orally vaccinated with *S.* Typhimurium expressing NrdF encoded by a prokaryotic expression plasmid failed to produce a serum or secretory antibody response specific to NrdF, and IFN-γ was not produced [80]. In another study conducted by Chen and colleagues [81], a RASV of *S*. Typhimurium was used, and the gene of interest was cloned into both eukaryotic and prokaryotic expression vectors. Immunogenicity was assessed in mice orally immunized with *M. hyopneumoniae* P97R1 adhesin, which induced specific Th1 cellular immune responses in a mouse model. However, no mucosal antibody responses against P97R1 were observed.

In the delivery system of *S.* Typhimurium expressing important fimbriae of *Escherichia coli* F4 (K88), F5 (K99), F6 (987Ps), and F41 and intimin adhesin, using a murine model with a single dose or double dose of 2 × 10^9^ CFU in 20 µL, IgG and IgA titers for individual adhesins in all immunized groups were higher in the booster dose group than in the single dose group [64]. In another study, Hur, Stein, and Lee [65] also used live-attenuated *S.* Typhimurium expressing other recombinant *E. coli* fimbrial antigens K88ab, K88ac, FedA, and FedF. The IgG2a titer was increased in the one-dose group, whereas both the IgG2a and IgG1 titers were increased in the two-dose group. Furthermore, vaccine strains were not detected in the feces excreted from immunized mice. Hur and Lee [61] evaluated the immune responses of various doses of *Salmonella* ghost (non-living, devoid of cytoplasmic content, maintaining their cellular morphology), with controlled expression of the φX174 E lysis gene being achieved in pigs. These bacterial ghosts carried enterotoxigenic *E. coli* fimbrial antigens (ETEC) to protect against colibacillosis in piglets. All groups were orally immunized with doses of 2 × 10^9^, 2 × 10^10^, or 2 × 10^11^ CFU in 10 mL PBS and boosted at weeks 11 and 14 of pregnancy. The serum levels of immunoglobulin IgG, IgG, and IgA in the colostrum of sows in groups that received 2 × 10^10^ or 2 × 10^11^ CFU were significantly higher than those of sows in the control group. Notably, after a challenge with wild-type ETEC, neither piglet diarrhea nor mortality were observed.

In another study [85], live-attenuated *S.* Typhimurium JOL912, which contains the genes encoding P fimbriae (the *pap* gene cluster), the iron-regulated aerobactin receptor *iut*A, and CS31A surface antigen adhesin from avian pathogenic *E. coli* (APEC), was evaluated as the vaccine against APEC infection in chickens. The vaccine was administered orally, intramuscularly, or subcutaneously, using different doses according to three groups: a no-vaccine group, a single-vaccine-dose group, and another that received primary and booster immunizations. The birds were exposed to an intra-air sac challenge using a virulent APEC strain at a dose of 10^7^ UFC, and the group that received two vaccine doses showed greater protection against the challenge (80%). Furthermore, this group showed significant increases in plasma IgG levels at three and for weeks old compared to birds from other groups, reinforcing the use of two vaccine doses [85]. Lee and colleagues [86] reported that, following a challenge with a virulent APEC strain, there were no deaths in the vaccinated group, whereas the control group showed a mortality rate of 15%. The administration of primary and booster doses of the *Salmonella*-delivered APEC vaccine candidate significantly enhanced the generation of antigen-specific sIgA, as well as the production of IFN-γ, IL-6, and IL-2, thereby providing protection for chickens against colibacillosis [86]. In another study, Oh et al. [88] used the P fimbria subunit PapA from APEC, which was in live-attenuated *S.* Typhimurium. Furthermore, the study utilized the non-toxic B subunits of cholera toxin (CTB) and heat-labile toxin (LTB) as adjuvants within the vaccine formulation. Mice were inoculated with 20 µL containing 2 × 10^9^ CFU. The findings demonstrated a significant increase in Pa-pA-specific serum IgG and mucosal IgA titers when the recombinant *Salmonella* vaccine was administered in conjunction with LTB or CTB adjuvants, highlighting the enhanced immunogenic response facilitated by these adjuvants. Rapid declines in immune responses throughout the experimental period were observed in mice immunized without an adjuvant.

The benefits of the intracellular action of *Salmonella* also correlate with natural infection in mucous membranes and in the intestinal tract, an analogous route to one of the most important infection routes of *Brucella abortus*. In a study by Kim et al. [29], live-attenuated *S.* Typhimurium was engineered to express BCSP31, Omp3b, and SOD proteins from *B. abortus*. Mice were vaccinated with a mixture of the three strains, either orally or intramuscularly [29]. The recombinant vaccine induced serum concentrations of IgG, TNF-α, and IFN-γ that were higher than the control when administered via the oral route (except Omp3b) and intramuscularly. A robust IFN-γ-mediated response helps eliminate *Brucella* infection in the host. Furthermore, it was found that after a challenge with a virulent strain of *B. abortus*, the vaccine was able to limit the colonization of the bacteria in the spleen of mice.

Another study targeting brucellosis in goats, reported by Leya and colleagues [24], developed a *S.* Typhimurium vaccine expressing four (BLS, PrpA, Omp19, and SOD) heterologous *Brucella* antigens and inoculated them subcutaneously with two doses: 5 × 10^9^ CFU/mL (Group B) and 5 × 10^10^ CFU/mL (Group C). The goats were challenged with a virulent *B. abortus* strain six weeks after immunization. Serum IgG titers against specific antigens in goats from Group C were significantly higher than those in non-immunized goats and the vector control group. Following antigenic stimulation, IFN-γ levels in peripheral blood mononuclear cells were significantly elevated in Groups B and C compared to the vector control group. The immunized goats in Group C, which received the highest dose, exhibited a significantly higher level of protection (*p* < 0.05); however, the group with the lower dose also showed a successful reduction in microgranuloma lesions in the liver induced by *B. abortus* infection. Stabel et al. [103] reported that the use of the *Salmonella* Cholerasuis chi 3781 (SC) strain expressing the BCSP31 protein from *B. abortus*, when administered orally to pigs and mice, stimulated a strong serum IgG response to both the recombinant protein and SC in mice. In contrast, orally inoculated pigs did not develop significant serum or intestinal antibody responses.

### 5.3. Recombinant Salmonella Expressing Virus Antigens

Live-attenuated strains of *Salmonella*, including *S.* Typhimurium, used in most of the studies reviewed in this article, but also *S.* Galinarium, *S.* Choleraesuis, and *S.* Pullorum, have been evaluated for use as live vaccines for the delivery of a variety of viral antigens (Table 2).

Among the targets studied were the hemagglutinin gene (HA1) from one of the avian influenza viruses (AIV or HPAI) of the H5N1 subtype. Liljebjelke and colleagues [108] reported the use of *S.* Typhimurium expressing AIV HA1 as an oral vaccine carrier in birds with doses of 10^9^ CFU. Animals were challenged with homologous A/whooper swan/Mongolia/3/2005-(CQ95) or heterologous A/Chicken/Queretaro/14588-19/95-(WM05) strains of the HPAI virus. Groups that received the recombinant vaccine demonstrated a statistically significant increase in survival compared with control groups (100%) for the low-dose homologous challenge with CQ95 and partial protection against the low-dose challenge with WM05. Neither vaccine provided protection to chickens when challenged with high doses of either HPAI virus, although survival was better against the challenge with CQ95 (60%). The presence of antibodies that recognize the HA protein in serum and probe samples was assessed by hemagglutination inhibition (HI) assay and collected 2 weeks after vaccination. Furthermore, Jazayeri and collaborators [110] reported the use of glycoproteins HA, NA, and NP from AIV expressed in live-attenuated *S.* Typhimurium SV4089, administered orally to birds using the same dose as the previous study. Fluorescence in situ hybridization (FISH) was used for detection, and *Salmonella* was specifically identified using the genus-specific probe Sal3 from homogenized sections of the spleen, liver, and cecum of infected chickens, where the distinct fluorescent signal of rod-shaped bacteria could be detected. They achieved the successful elimination of *Salmonella* from the spleen and liver of infected birds, but it was still detectable in the cecum even 35 days after inoculation, demonstrating that live-attenuated *S.* Typhimurium provides an alternative in terms of in vitro stability of the transfected plasmid.

In a study using subtype H9N2 avian influenza virus, two attenuated *S. Typhimurium* strains were constructed: one with the O antigen of LPS intact (smooth strain) and the other without it (rough strain), as the removal of this antigen can increase the immunogenicity of surface proteins. This experiment, conducted in an avian model with recombinant *Salmonella* expressing H9N2 hemagglutinin (HA), evaluated whether *Salmonella* could act as a transporter to enhance immune responses to delivered antigens. Both the S-HA and R-HA strains elicited similar HA-specific immune responses, as indicated by serum IgG and HI titers, suggesting that the deletion of the O antigen does not impact the immunogenicity and delivery properties of the *Salmonella* system [30]. Additionally, Hajam et al. [113] used *S.* Gallinarum (SG) for expressing HA1, HA2, and/or the conserved ectodomain of matrix protein 2 (M2e) in the development of H9N2 vaccine strains, which were compared to a commercially available oil-adjuvanted inactivated H9N2 full-virus vaccine in a chicken model [30,113]. In search of a cheaper vaccine, and without the use of exogenous adjuvants, the experiment vaccinated chickens with a single dose, orally at a dose of 10^9^ CFU, in groups who received either the individual H9N2 genes in SG, or as a mixture of these, while the control was vaccinated intramuscularly with the inactivated commercial H9N2 vaccine. An indirect ELISA of IgY with serum samples collected on days 14 and 28 post-vaccination showed that animals vaccinated with the RASV or a mixture of vaccine strains showed specific systemic responses to HA1 that were significantly higher (*p* < 0.05) than those of the control group. Cytokine gene expression revealed that IFN-γ increased by over four times (*p* < 0.05) in all groups inoculated with RASV constructs compared to the PBS control group. However, the commercial vaccine induced significantly greater responses for the HA1 and HA2 genes but was not as robust for M2 when compared to the RASV. Upon challenge, chickens immunized with both vaccines exhibited comparable lung inflammation and viral loads, although both were significantly lower than those in the group vaccinated with SG alone. However, immunization with the RASV managed to efficiently inhibit the infection and spread of H9N2.

Another use of orally administered vaccines has been to combat duck enteritis virus (DEV), an acute disease that affects ducks, geese, swans, and other free-living aquatic birds, with high mortality. Yu and colleagues [117] used live-attenuated *S.* Typhimurium (SL7207) with *E. coli* LTB as an adjuvant, fused to the DEV UL24 gene in ducks. Birds were orally inoculated with SL7207 (pVAX-UL24) or SL7207 (pVAX-LTB-UL24) with 1 × 10^10^ CFU. Immunization of animals with the recombinant LTB vaccine showed superior protective efficacy (60–80%) against a lethal DEV challenge, compared to the limited survival rate (40%) of those immunized with the vaccine without the adjuvant. To support this study’s results, Liu et al. [118] orally immunized ducks with *S.* Typhimurium S739 expressing DEV genes and adjuvants (LTB subunit and duck DuIL-2 gene). After a booster immunization, 90% of ducks immunized with recombinant *Salmonella* and the LTB adjuvant were protected during lethal challenge. IgY levels were slightly higher against the tUL24 protein in ducks vaccinated with UL24-LTB and UL24-DuIL-2 on days 10, 21, and 28 post-immunization (*p* < 0.05). Serum IgY and bile IgA levels in response to purified DEV were slightly lower than those for tUL24, but higher IgY titers against DEV were observed in ducks vaccinated with UL24 and tgB compared to those for tUL24 (*p* < 0.05). Among all groups, the highest bile IgA levels were seen in ducks receiving the attenuated *Salmonella*-DEV DNA recombinant vaccine [118]. Although the two adjuvants stimulated a high immune response in ducks, the vaccination with recombinant *Salmonella* and DuIL-2 was not capable of providing protection against homologous challenge.

Cytokines like IFN-α help modulate innate and adaptive immunity, providing a first line of defense against viral infections. However, their use in livestock is costly. Kim et al. [122] tested an oral vaccine using live-attenuated *S.* Typhimurium engineered to secrete porcine IFN-α (swIFN-α) to prevent clinical signs of transmissible gastroenteritis virus (TGEV), a significant economic threat in the swine industry. Administered at doses of 10^9^ and 10^11^ CFU per pig, the vaccine effectively reduced the severity of TGEV-induced clinical signs [122]. To assess the virus’s spread in piglets infected with TGEV, the quantity of TGEV in fecal samples collected from the infected piglets was measured. Virus shedding was detected one day after TGEV infection and reached its peak at four days post-infection. However, piglets that received the recombinant vaccine (at doses of 10^9^ and 10^11^ UFC) exhibited reduced viral shedding at four days post-infection. Likewise, the amount of TGEV was lower in the intestinal tissues and mesenteric lymph nodes of piglets inoculated with the recombinant vaccine when compared to the control, helping to reduce the severity of clinical signs caused by TGEV infection.

In another study on TGEV, Zhang and collaborators [123] sought to evaluate an experimental vaccine delivered by live-attenuated *S.* Typhimurium expressing the structural protein of the virus, which is correlated with another virus that causes swine epidemic diarrhea (PEDV). These viruses are members of the Coronaviridae family, and both viruses can cause severe enteropathogenic diarrhea in pigs; therefore, the simultaneous induction of immune responses is promising for the food industry. Piglets were immunized orally with recombinant *Salmonella* at a dosage of 1.6 × 10^11^ CFU per piglet and then immunized with a booster of 2 × 10^11^ CFU. The RASV with two S proteins from TGEV and PEDV simultaneously stimulated immune responses against both viruses after oral immunization. Antibody levels against PEDV or TGEV in piglets immunized with the RASV of *S*. Typhimurium began to increase at 2 weeks, but the difference compared to controls was not statistically significant until the sixth week. Serum IgG levels against PEDV and TGEV were significantly higher (*p* < 0.01) in piglets immunized with the recombinant vaccine than with PBS or empty vector from weeks 4 to 8. Significantly elevated levels of IgG and IgA antibodies against PEDV and TGEV were induced by the RASV in week 6, though these levels were slightly lower than those induced by the monogenic vaccine and empty vector. The results showed that T lymphocyte proliferation levels increased to a statistically significant level compared to the control group in weeks 4 to 6, being higher in piglets immunized with the RASV when compared to other vaccine groups, but no significant differences were observed (*p* > 0.05). The results also indicated that IFN-γ and IL-4 levels in piglets treated with the RASV were significantly higher (*p* < 0.01) than in control groups.

### 5.4. Recombinant Salmonella Expressing Parasite Antigens

The protective mechanisms required to combat parasites differ significantly from those required for other pathogens, and parasites can actively suppress the host’s immune response [133]. This has made it challenging to identify an effective combination of antigens, adjuvants, and routes of administration for vaccination [133,134]. However, recent years have seen notable advances in the development of vaccines utilizing recombinant antigens from these parasites, although studies in this area remain limited [134].

In this review, we compiled 14 articles (Table 3) that investigate the use of attenuated strains of *Salmonella* engineered to express parasite antigens. These attenuated strains are particularly attractive as live vectors because they can elicit strong mucosal immunity, which is crucial for controlling certain parasites, such as *Trichinella spiralis*, in the intestinal mucosa [134].

Pompa-Mera and collaborators [31] used *S.* Typhimurium SL3261 and inserted a fusion glycoprotein from *T. spiralis* larvae. The vaccine was administered intranasally at a dose of 1× 10^8^ CFU to BALB/c mice. After challenge, mice immunized intranasally with recombinant *Salmonella* saw a reduction in the parasite load of adult *T. spiralis* by 61.83% on the eighth day post-infection, indicating a protective immune response. This immune response was characterized by the induction of antigen-specific IgG1 and IL-5 production. In another study using *T. spiralis*, the Ts87 gene was attenuated to strain *S.* Typhimurium SL7207, administered only orally to mice. They also reported a statistically significant 29.8% reduction in adult worm burden and a 34.2% reduction in larvae following *T. Spiralis* larvae challenge, compared with mice immunized with empty *Salmonella* or a PBS control. However, mice that received the recombinant *Salmonella* vaccine exhibited elevated levels of IgG2a and IgG1 subclass antibodies, with no significant difference (*p* > 0.05) between IgG2a and IgG1 levels, indicating a mixed Th1/Th2 immune response. Additionally, there was a notable increase (*p* < 0.05) in total intestinal IgA levels among mice immunized with the recombinant vaccine compared to those in the vector or PBS-only groups. Another important parasite for veterinary medicine is the cestode *Echinococcus granulosus* (EgDf1), which infects the intestines of dogs, in addition to having intermediate hosts such as herbivorous and omnivorous animals and, accidentally, humans. Chabalgoity and colleagues [139] produced a vaccine in which fatty-acid-binding proteins (FABPs) of EgDf1 fused with a C-terminal fragment of tetanus toxin (TetC) were expressed in *S.* Typhimurium LVR01. The inoculation was administered intravenously with a dose of 10^6^ CFU, as well as via an oral dose of 4 × 10^9^ CFU, in mice, eliciting an antibody response to EgDf1, the production of Th1-related antigen-specific cytokines, and significant levels of a Th2 cytokine protein in the spleen cells of orally immunized mice. Furthermore, sera from immune mice reacted strongly with fixed sections of the larval stage of the worm. Another study by the group used *S.* Typhimurium LVR01 expressing EgDf1 FABP in dogs, which were orally vaccinated at a dose of 5 × 10^10^ CFU in 2 mL of PBS or given PBS alone. The dogs presented IgG antibody responses against EgDf1 when immunized with LVR01 (pTECH ± EgDf1). All animals developed high titers of IgG antibodies against LPS in serum by week 4 after a single dose of the recombinant vaccine [48].

Cong et al. [73] tested a vaccine using live-attenuated *S.* Typhimurium as a vector for the recombinant plasmid pSAG1-2/CTA2/B, which encodes *Toxoplasma gondii* antigens SAG1 and SAG2 linked to cholera toxin subunits (CTA2/B). Orally administered in mice, this vaccine induced anti-*T. gondii* IgG antibodies, with increased IgG levels in mice that received CTA2/B as a genetic adjuvant compared to controls (*p* = 0.003, *p* = 0.004). Although both groups generated anti-*T. gondii* antibodies, mice vaccinated with CTA2/B showed a predominant Th1 response, while those without it exhibited a Th2 response. Upon challenge with virulent *T. gondii*, mice vaccinated with CTA2/B had longer survival times and a 40% survival rate (*p* = 0.003) [73].

In another study, Cong et al. [22] developed a live-attenuated *S.* Typhimurium vaccine encoding *T. gondii* epitopes (SAG1, GRA1, ROP2, GRA4, SAG2C, SAG2X) linked to CTA2/B, which was delivered to BALB/c mice via oral, nasal, or intramuscular routes. Mice immunized orally and nasally showed higher anti-*T. gondii* antibody levels than those vaccinated intramuscularly (*p* < 0.05). Flow cytometry revealed CD4+ and CD8+ T-cell activation, with intramuscular and intranasal immunizations inducing 28.54% and 30.01% activation, respectively. IFN-γ and IL-2 levels were significantly higher in orally and nasally vaccinated groups than in the control (*p* = 0.02), while IL-4 and IL-5 remained low across groups. Antigen-specific lymphocyte proliferation was also higher in the oral and nasal groups. After a lethal *T. gondii* challenge, survival rates were 20% for intramuscular, 40% for intranasal, and 60% for oral immunization. These results highlight the potential of oral and nasal routes for delivering live-attenuated *Salmonella* vaccines in inducing stronger immune responses and improved protection against *T. gondii* [22].

Benitez, McNair, and Mead [74] utilized strains of live-attenuated *S.* Typhimurium expressing Cp23 and Cp40 from *Cryptosporidium parvum*, which are recognized as surface immunodominant antigens, as they are recognized by serum antibodies from humans and various animal species. In the study, mice received an oral immunization of 5 × 10^9^ CFU per mouse and an intragastrical immunization of 0.2 mL of PBS for each vaccine, which included a vector with an empty plasmid, the RASV vaccine expressing the Cp23 gene, and another for the CP40 gene. Two booster doses, consisting of 100 μg of RASV, were injected subcutaneously on days 0 and 14, followed by oral immunization against *Salmonella*. The production of IgG and IgG1 subclasses was observed in vaccinated mice after 7 weeks of immunization. The specific serum levels of anti-Cp23 and anti-Cp40 IgG were significantly increased in mice immunized with the RASV vaccine compared to mice immunized with the control vector. IgA titers were detected in mice immunized with the RASV expressing Cp23 but not in animals immunized with the Cp40 construct (*p* > 0.05). Only an IgG1 antibody response was obtained, with no IgG2a response, suggesting a Th2-type response was elicited.

Chen et al. [142] studied a live-attenuated *S.* Typhimurium vaccine using active promoters (nirB, pagC, or pMohly) to express the *Schistosoma japonicum* antigen Sj23LHD-GST via the *Salmonella* type III secretion system or α-hemolysin. Mice were orally immunized with 0.2 mL PBS containing 10^9^ CFUs of either recombinant *S.* Typhimurium or an empty vector, with a PBS-only group as a control. After three doses, mice vaccinated with the nirB promoter showed a moderate IgG response and the highest IgG2a ratio, indicating a strong Th1-type response. CD44 expression in splenocytes was significantly elevated in mice that received the nirB-driven antigen (25 ± 2%) compared to other groups (*p* < 0.01). Following *S. japonicum* challenge, mice immunized with *S.* Typhimurium containing nirB, pagC, or pMohly1 promoters showed egg burden reductions of 57.71%, 30.07%, and 40.46%, respectively. The nirB-driven antigen delivered by type III secretion reduced parasite burden by 51.35% and egg burden by 62.59%, showing promising in vivo protective efficacy. These findings highlight the protective efficacy of antigens delivered by the *Salmonella* type III secretion system using the nirB promoter [142].

## 6. Conclusions and Future Directions

Developing attenuated vaccines requires balancing safety and immunogenicity. Although *Salmonella* shows promise as a vaccine vector, many studies have not achieved significant protective efficacy. Over-attenuation can reduce immunogenicity and tissue colonization, while insufficient attenuation may pose biosafety risks. While safety is often evaluated based on the absence of adverse reactions and strain stability, few studies assess environmental release or potential transmission through vertical or horizontal pathways.

The development and commercialization of RASVs represents a significant advancement in veterinary medicine. These vaccines offer a safe and effective means of controlling infectious diseases in livestock, reducing the reliance on antibiotics and mitigating the risks associated with antibiotic resistance. The ability of RASVs to deliver antigens from a wide range of pathogens makes them versatile tools for disease prevention.

Looking forward, future research should focus on optimizing vaccine formulations and delivery methods, particularly for ruminants and other species where oral administration remains a challenge. Additionally, there is potential to expand the use of RASVs beyond veterinary applications, with possible implications for human medicine, particularly in the context of zoonotic diseases.

The studies reviewed here highlight the promise of RASVs as part of an integrated approach to managing infectious diseases in animal populations. Continued investment in research and development will be essential to fully realize the potential of these vaccines and to address the challenges that remain.

## Figures and Tables

**Table 1:** Bacterial antigens expressed in *Salmonella*.

Antigen (Organism)	*Salmonella* Strain or Plasmid	Attenuation	Route/Dose (CFU)	Model	Immunity	Reference
SspH2, LipL32 (*Leptospira* spp.)	*S.* Typhimurium SL3261	Δ*aroA*	O/1 × 10^7^	Rat	HI, MI, CI	[55]
NrdF (*M. hyopneumoniae*)	*S.* Typhimurium SL3261	Δ*aroA*	O/10^9^	Mice	HI, MI	[78]
	*S.* Typhimurium SL3261	Δ*aroA*	O/1 × 10^9^	Swine	HI, MI, CI	[79]
	*S.* Typhimurium CS332	Δ*aroA*	O/2 × 10^8^; 2nd dose 3 × 10^8^	Mice	HI, MI, CI	[80]
P97R1 (*M. hyopneumoniae*)	*S.* Typhimurium CS332	Δ*aroA*	O/2 × 10^8^; 2nd dose 3 × 10^8^	Mice	HI, MI, CI	[81]
K88ab (*Escherichia coli*)	*S.* Typhimurium G30/pFM205	*galE*	O, IP/1 × 10^8^	Mice	HI	[82]
	*S.* Typhimurium	Δ*lon* Δ*cpxR* Δ*asd*	O/1 × 10^8^	Mice	HI, MI	[65]
	*S.* Typhimurium	Δ*lon* Δ*cpxR* Δ*asd*	O/2 × 10^10^	Pregnant sows and piglets	HI, MI	[64]
K88ab, K88ac, K99, FasA, F41 (*E. coli*)	*S.* ghost-controlled expression of φX174 lysis gene E		O/primed and boosted 2 × 10^9^, 2 × 10^10^, and 2 × 10^11^	Pregnant sows and piglets	HI, MI	[61]
	*S.* Typhimurium JOL912	Δ*lon*, Δ*cpxR*, Δ*asd*	IM/1 × 10^8^	Mice	HI, CI, MI	[83]
Stx2eB, FedF, FedA F18+ Shiga toxin (*E. coli*)	*S.* Typhimurium JOL1311 and JOL912	Δ*asd*, Δ*lon*, Δ*cpxR*	IM/9 × 10^7^	Mice	HI, CI, MI	[84]
fliC F18+ Shiga toxin (*E. coli*)	*S.* Typhimurium JOL1454, JOL1460, JOL1464	Δ*lon*, Δ*cpxR*, Δ*asd*	SC/3 × 10^7^	Mice	HI, CI, MI	[27]
APEC papA, papG, iutA, and clpG (*E. coli*)	*S.* Typhimurium JOL912	Δ*lon*, Δ*cpxR*, Δ*asdA16*	O/1 × 10^7^	Chicken	HI, CI, MI	[85]
APEC papA, papG, iutA, and clpG (*E. coli*)	*S.* Typhimurium JOL912	Δ*lon*, Δ*cpxR*, Δ*asdA16*	O/1 x 10^7^	Chicken	HI, CI, MI	[86]
APEC O-antigen (*E. coli*)	*S.* Typhimurium S100	Δ*asd*, Δ*crp*, Δ*cya*, Δ*rfbP*	O/× 10^9^, IM/5.0 × 10^7^	Chicken	HI, MI	[87]
APEC PapA, CTB and LTB (*E. coli*)	*S.* Typhimurium *χ*8501	*hisG*, Δ*crp-28*, Δ*asdA16*	O/2 × 10^9^	Mice	HI, MI	[88]
APEC (*E. coli*)	*S.* Typhimurium *χ*8025	Δ*asd*	O/1 × 10^8^	Chicken	MI	[89]
tHP (*Clostridium perfringens*)	*S.* Typhimurium	Δ*asd*	O/1 × 10^9^	Chicken	Intestinal colonization, BSG	[66]
tHP (*C. perfringens*)	*S.* Typhimurium *χ*9352	Δ*asd*, *lacI*	O/1.2 × 10^9^	Chicken	MI	[90]
α-toxin, NetB toxin, Fba (*C. perfringens*)	*S.* Typhimurium *χ*11802	Δ*asd*, *lacI*	O/1 × 10^8^ or 1 × 10^9^	Chicken	CI, MI	[91]
PLcC, GST-NetB (*C. perfringens*)	*Salmonella* vaccine *(PIESV) χ*11802 and *χ*12341	*asdA*, *murA*	O/~5 × 10^8^	Chicken	NE Intestinal Lesion Scoring	[33]
O antigen (*Burkholderia mallei*)	*S.* Typhimurium SL326	Δ*aroA*	IN/1 × 10^7^	Mice	HI, MI	[68]
M protein (*Streptococcus pyogenes*)	*S.* Typhimurium *LB5000*	*-*	SC/Rabbit: 10^8^ heat-killed bacteria or purified flagella; IP/Mice: 1 × 10^6^ to 2 × 10^6^ live vaccine	Mice and Rabbit	HI	[92]
optA, optB, LfliC, Lhly (*Lawsonia intracellularis*)	*S.* Typhimurium JOL912	Δ*asd*	O/1 × 10^7^	Mice	HI, MI	[93]
Sip (*Streptococcus agalactiae*)	*S.* Typhimurium SL7207	Δ*aroA*	IG/10^7^, 10^8^ and 10^9^	Fish	HI	[32]
F1, I2 (*Pseudomonas aeruginosa*)	*S.* Typhimurium LH430	*phoP/phoQ*,Δ*asd*	O and SC/2.0 × 10^8^ to 2.0 × 10^10^	Mice	HI, CI, MI	[75]
CP39, FimA, PtfA, ToxA (*Pasteurella multocida*) F1P2 (*Bordetella bronchiseptica*)	*S.* Typhimurium JOL912	Δ*lon*, Δ*cpxR*, Δ*asd*	IN/1 × 10^5^	Mice	HI, MI	[69]
CjaA (*Campylobacter jejuni*)	*S.* Typhimurium LB5010	Δ*aroA*, *fliM*, *spaS*, *ssaU*	O/1 × 10^8^	Chicken	HI, MI	[94]
CjaA (*C. jejuni*)	*S.* Typhimurium *χ*9718	Δ*asd*	O/1 × 10^8^	Chicken	MI	[95]
BCSP31 (*Brucella abortus*)	*S.* Typhimurium chi 4064	Δ*cya*, Δ*crp*	O/2 × 10^8^ to 4 × 10^8^	Mice	HI, MI, Blatogenisis	[96]
BCSP31 (*B. abortus*)	*S.* Typhimurium chi 4064	Δ*cya*, Δ*crp*	O/1 × 10^10^ to 2 × 10^10^	Crossbred swine	HI, MI, Blatogenisis	[97]
L7/L12, BLS (*B. abortus*)	*S.* Typhimurium X4072	Δ*asd*	O/1 × 10^9^	Mice	HI, CI, MI	[98]
BCSP31, Omp3b, SOD (*B. abortus*)	*S.* Typhimurium JOL912	Δ*lon*, Δ*cpxR*, Δ*asd*	IP/1.2 × 10^6^; O/1.2 × 10^9^	Mice	HI, CI	[29]
SOD, BLS, PrpA, Omp19 (*B. abortus*)	*S.* Typhimurium JOL912 and JOL1800	Δ*lon*, Δ*cpxR*, Δ*asd*	O and IP/2 × 10^7^	Mice	CI, MI	[77]
BCSP31, Omp3b, SOD (*B. abortus*)	*S.* Typhimurium JOL911 and JOL912	Δ*lon*, Δ*cpxR*, Δ*asd*	IP/1.2 × 10^4^, 1.2 × 10^5^ and 1.2 × 10^6^	Mice	HI, CI	[99]
BCSP31, Omp3b, and SOD (*B. abortus*)	*S.* Typhimurium pMMP65	Δ*lon*, Δ*cpxR*, Δ*asd*	SC/3 × 10^9^	Dog	HI, CI	[100]
PrpA (*B. abortus*)	*S.* Typhimurium JOL1818 and JOL1881	Δ*lon*, Δ*cpxR*, Δ*asd*, Δ*rfaL*	IP/1 × 10^7^	Mice	HI, CI	[77]
SOD, BLS, PrpA, Omp19 (*B. abortus*)	*S.* Typhimurium JOL1800	∆*lon*, ∆*cpxR*, ∆*asd*	SC/5 × 10^9^ and 5 × 10^10^	Goat	HI, CI	[24]
BCSP31, Omp3b, and SOD (*B. abortus*)	*S.* Typhimurium JOL912	Δ*lon*, Δ*cpxR*, Δ*asd*	SC/3 × 10^9^	Goat	HI, CI	[101]
L7/L12 (*B. abortus*)	*S.* Typhimurium JOL1800	∆*lon*, ∆*cpxR*, ∆*asd*, ∆*rfaL*	IM/10^7^	Mice	HI, MI	[102]
BCSP31 (*B. abortus*)	*S.* Choleraesuis chi 3781	∆*cpxR*, ∆*cya*	O/Mice: 4 ×10^10^; Swine: 4 × 10^8^ to 6 × 10^8^	Mice and crossbred swine	HI, MI,	[103]
6-PGD (*Streptococcus sui*s)	*S.* Choleraesuis rSC0011	Δ*asd*	O/1 ± 0.3 × 10^9^	Mice	HI, MI	[104]
Serotypes 2 and 7 (*S. sui*s)	*S.* Choleraesuis rSC0016	Δ*sopB*	O/Suis: 1 ± 0.3 × 10^9^; Mice: 1 ± 0.3 × 10^9^	Mice and Swine	HI, MI	[28]
SaoA (*S. sui*s)	*S.* Choleraesuis rSC0012	Δ*fur*	O/1 ± 0.2 × 10^9^	Mice	HI, MI, CI	[105]
Serotypes 1/2, 2, 3, 7, 9 (*S. sui*s)	*S.* Choleraesuis rSC0016	Δ*sopB*, Δ*asd, lacl*	O/1 ± 0.2 × 10^9^	Mice	HI, CI	[106]
P42, P97 (*M. hyopneumoniae*)	*S.* Choleraesuis rSC0016	Δ*asd*	O/10^9^	Mice	HI, MI, CI	[107]
F18+ Shiga toxin (*E. coli*)	*S.* Choleraesuis C520	*crp,* Δ*asd*	O/2 × 10^9^	Swine	HI, MI, CI	[67]

IG, intragastric; IM, intramuscular; IN, intranasal; IP, intraperitoneal; O, oral; SC, subcutaneous; CFU, colony-forming unit; HI, humoral immunity; CI, cellular immunity; MI, mucosal immunity.

**Table 2:** Viral antigens expressed in *Salmonella*.

Antigen (Organism)	*Salmonella* Strain or Plasmid	Attenuation	Route/Dose (CFU)	Model	Immunity	Reference
HA (AIV H5N1)	*S.* Typhimurium BRD509	Δ*aroA*, Δ*aroD*	O/10^9^	Chicken	Hemagglutination inhibition	[108]
chIFN-a, chIL-18 (AIV H9N2)	*S.* Typhimurium *χ*8501	*hisG*, Δ*crp-28*, Δ*asdA16*	O/10^9^ and 10^11^	Chicken	CI, hemagglutination inhibition, PCR	[109]
HA, NA, NP (AIV H5N1)	*S.* Typhimurium SV4089	*Dam*, Δ*PhoP*	O/10^9^	Chicken	PCR, FISH, and culturing on XLT4	[110]
HA (AIV H5N1)	*S.* Typhimurium SV4089	*Dam*, Δ*PhoP*	O, IM/10^9^	Chicken	CI, hemagglutination inhibition, PCR	[111]
HA (AIV H9N2)	*S. Typhimurium* JOL912, JOL1800	Δ*lon*, Δ*cpxR*, Δ*asd*	O/10^8^	Chicken	HI, hemagglutination inhibition	[30]
HA (AIV H7N1)	*S.* Typhimurium JOL1863	Δ*lon*, Δ*cpxR*, Δ*asd*	O, IN, IM/10^9^	Chicken	HI, MI, Hemagglutination inhibition	[70]
HA, M2, NA (LPAI H7N9)	*S.* Typhimurium JOL1800	O antigen deficient	O/10^9^	Chicken	HI, CI, MI	[112]
H9N2 haemagglutinin, M2 (AIV H9N2)	*S.* Gallinarum JOL967	Δ*lon*, Δ*cpxR*, Δ*asd*	O, IM/10^9^	Chicken	HI, CI, MI	[113]
swIFN-α, swIL-18 (TGEV)	*S.* Typhimurium 8501	*hisG*, Δ*crp-28*, Δ*asdA16*	O/10^11^	Swine	Gross lesion, histopathology, qRT-PCR	[114]
Glycoprotein B (PrV)	*S.* Typhimurium SL7207	Δ*aroA*	O/5 to 10 10^7^	Mice	HI, MI	[115]
swIL-18, swIFN-α (PrV)	*S.* Typhimurium *χ*8501	*hisG*, Δ*crp-28*, Δ*asdA16*	O/10^11^	Swine	HI, CI	[116]
UL24 (DEV)	*S.* Typhimurium SL7207	*hisG46*, *DEL407*, Δ*aroA*	O/10^11^, 10^10^ or 10^9^	Duck	HI, CI, MI	[117]
tgB, UL24 (DEV)	*S.* Typhimurium *S*739	Δ*asd-66*, Δ*crp-24*, Δ*cya-25*	O/10^10^; 10^11^ or 10^12^	Duck	MI	[118]
CD2v/CTL/9GL, p54/p12/p72(ASFV)	*S.* Typhimurium JOL912	Δ*lon*, Δ*cpxR*, Δ*asd*	IM/10^8^	Swine	HI, CI, MI	[119]
S1, N (IBV)	*S.* Typhimurium SL7207	Δ*aroA*	O, IN/1 × 10^9^, 5 × 10^9^ or 1 × 10^10^	Chicken	HI, MI	[71]
VP2/4/3 (IBVD)	*S.* Typhimurium	*Dam*, *Phop*	O/10^9^, 10^8^ or 10^7^	Chicken	HI	[120]
prM-E (TMUV)	*S.* Typhimurium *SL7207 + adenovirus adjuvant with duck IL-2*	Δ*aroA*	O, IM/10^7^, 10^10^	Duck	HI, CI	[121]
N (TGEV)	*S.* Typhimurium SL7207	Δ*aroA*	IG/10^7^, 10^8^ or 10^9^	Mice	HI, MI	[72]
swIFN-α (TGEV)	*S.* Typhimurium *χ*8501	*hisG*, Δ*crp-28*, Δ*asdA16*	O/10^9^ or 10^11^	Swine	qRT-PCR	[122]
S (TGEV, PEDV)	*S.* Typhimurium SL7207	Δ*aroA*	O/1.6 × 10^11^	Swine	HI, CI, MI	[123]
N (TGEV)	*S.* Typhimurium SL7207	Δ*aroA*	O/10^12^	Swine	HI, CI, MI	[124]
M (TGEV)	*S.* Typhimurium *m* SL7207	Δ*aroA*	IG/10^9^	Mice	HI, CI, MI	[125]
Glycoprotein 5, TLR-5 (PRRSV)	*S.* Typhimurium SL7207, FljB		IP/50 μg	Mice	HI	[126]
VP1 (FMDV)	*S.* Typhimurium KST0666	*Irradiated*	IP/1 × 10^4^ to 3 × 10^8^	Mice	HI, CI, MI, VN	[127]
p27 capsid (SIV)	*S.* Typhimurium PV4570	Δ*aroA*	IM, IG/10^10^	Rhesus macaques	HI, CI, MI	[128]
Glycoprotein (RV), LTB (*E. coli*)	*S.* Typhimurium LH430	*phoP*, *phoQ*	O/5 × 10^10^	Mice	HI, CI	[129]
siRNA expressing 3D, VP4 and 2B (FMDV)	*S.* Choleraesuis C500		IM/Guinea pigs: 1.0 × 10^9^; Swines: 5 × 10^9^	Guinea Pigs, Swine	SPB-ELISA	[130]
Cap (PCV2)	*S.* Choleraesuis rSC0016	Δ*sopB*, Δ*asdA*	O/10^9^	Mice	HI, CI, MI, qPCR, VN	[28]
HN (NDV)	*S.* Pullorum C79-13	Δ*crp*, Δ*asd*	O/10^9^	Chicken	HI, MI, hemagglutination inhibition	[131]
S1 (IBV)	*S.* Gallinarum JOL2068, JOL2077	Δ*lon*, Δ*cpxR*, Δ*asd*	O/10^9^	Chicken	HI, MI	[23]
M2e, CD154 (AIV H5N1)	*Salmonella enteritidis*	Δ*aroA*, Δ*htrA*	O/10^6^ to 10^8^	Chicken	MI, hemagglutination inhibition	[132]

IG, intragastric; IM, intramuscular; IN, intranasal; IP, intraperitoneal; O, oral; CFU, colony-forming unit; HI, humoral immunity; CI, cellular immunity; MI, mucosal immunity; VN, virus neutralization assays; AIV, avian influenza viruses; ASFV, African swine fever virus; DEV, duck enteritis virus; FMDV, foot-and-mouth disease virus; IBV, infectious bronchitis virus; IBVD, infectious bursal disease virus; LTB, heat-labile enterotoxin B; NDV, Newcastle disease virus; PCV2, porcine circovirus type 2; PRRSV, porcine reproductive and respiratory syndrome; PrV, pseudorabies virus; p3D-NT56, siRNA directed against the polymerase gene 3D of FMDV; SIV, simian immunodeficiency virus; TGEV, porcine transmissible gastroenteritis virus; TLR-5, toll-like receptor 5 TMUV, Tembusu virus; VLP, virus-like particles.

**Table 3:** Parasitic antigens expressed in *Salmonella*.

Antigen (Organism)	*Salmonella* Strain or Plasmid	Attenuation	Route/Dose (CFU)	Model	Immunity	Reference
Ts87 (*Trichinella spiralis*)	*S.* Typhimurium SL7207	Δ*aroA*	O/10^8^	Mice	HI, CI, MI	[135]
Ag30 (*T. spiralis*)	*S.* Typhimurium SL3261	Δ*aroA*	IN/10^9^	Mice	HI, CI, MI	[31]
TsNd (*T. spiralis*)	*S.* Typhimurium SL1344	Δ*cya*	O/10^8^	Mice	HI, CI, MI	[136]
DNase II (*T. spiralis*)	*S.* Typhimurium SL1344	Δ*cya*	O/10^8^	Mice	HI, CI, MI	[137]
rTsSP1.2 (*T. spiralis*)	*S.* Typhimurium SL1344	Δ*cya*	O/10^8^	Mice	HI, CI, MI	[138]
FABP (*Echinococcus granulosus*)	*S.* Typhimurium SL3261	Δ*aroA*	IV/10^6^, O/4 × 10^9^	Mice	HI, CI, MI	[139]
FABP (*E. granulosus*)	*S.* Typhimurium LVR01	Δ*aroC*	O/5 × 10^10^	Dog	HI, CI, MI	[48]
EmGAPDH (*Echinococcus multilocularis*)	*S.* Typhimurium		O/2 × 10^10^ or IP: 5 × 10^5^	Mice	Western blotting	[140]
gp63 (*Leishmania major*)	*S.* Typhimurium BRD509	Δ*aroA*, Δ*aroD*	O/1 × 10^10^	Mice	HI, CI	[141]
SAG, SAG2 (*Toxoplasma gondii*)	*S.* Typhimurium BRD509	Δ*aroA*, Δ*aroD*	IG/10^9^	Mice	HI, CI	[73]
Tachyzoite and bradyzoite proteins (*T. gondii*)	*S.* Typhimurium BRD509	Δ*aroA*, Δ*aroD*	O, IN, IM/1 to 5 × 10^9^	Mice	HI, CI, MI	[22]
Cp23, Cp40 (*Cryptosporidium parvum*)	*S.* Typhimurium SL3261 and LB5010	Δ*aroA*, *galE*	IG/5 × 10^9^	Mice	HI, MI	[74]
Sj23LHD-GST (*Schistosoma japonicum*)	*S.* Typhimurium VNP20009	*purI*, *msbB*	O/10^9^	Mice	HI, CI	[142]
EC-SOD (*Acanthocheilonema viteae*)	*S.* Typhimurium SL3261	Δ*aroA*	O/5 × 10^8^	Jird	HI	[143]

IG, intragastric; IM, intramuscular; IN, intranasal; IP, intraperitoneal; IV, intravenous; O, oral; CFU, colony-forming unit; HI, humoral immunity; CI, cellular immunity; MI, mucosal; EmGAPDH, immunityglyceraldehyde-3-phosphate dehydrogenase.

## Data Availability

No new data were created or analyzed in this study. Data sharing is not applicable to this article.
